# Effects of heat stress on growth performance, selected physiological and immunological parameters, caecal microflora, and meat quality in two broiler strains

**DOI:** 10.5713/ajas.19.0208

**Published:** 2019-07-01

**Authors:** Elmutaz Atta Awad, Muhamad Najaa, Zainool Abidin Zulaikha, Idrus Zulkifli, Abdoreza Farjam Soleimani

**Affiliations:** 1 Institute of Tropical Agriculture and Food Security, Universiti Putra Malaysia, 43400 UPM Serdang, Selangor, Malaysia; 2 Department of Poultry Production, University of Khartoum, 13314 Khartoum North, Sudan; 3 Department of Animal Science, Universiti Putra Malaysia, 43400 UPM Serdang, Selangor, Malaysia

**Keywords:** Broiler Chickens, Heat Exposure, Caecal Microbiota, α1-Acid Glycoprotein, Ceruloplasmin, Ovotransferrin

## Abstract

**Objective:**

This study was conducted to investigate the effects of normal and heat stress environments on growth performance and, selected physiological and immunological parameters, caecal microflora and meat quality in Cobb 500 and Ross 308 broilers.

**Methods:**

One-hundred-and-twenty male broiler chicks from each strain (one-day-old) were randomly assigned in groups of 10 to 24 battery cages. Ambient temperature on day (d) 1 was set at 32°C and gradually reduced to 23°C on d 21. From d 22 to 35, equal numbers of birds from each strain were exposed to a temperature of either 23°C throughout (normal) or 34°C for 6 h (heat stress).

**Results:**

From d 1 to 21, strain had no effect (p>0.05) on feed intake (FI), body weight gain (BWG), or the feed conversion ratio (FCR). Except for creatine kinase, no strain×temperature interactions were observed for all the parameters measured. Regardless of strain, heat exposure significantly (p<0.05) reduced FI and BWG (d 22 to 35 and 1 to 35), immunoglobulin Y (IgY) and IgM, while increased FCR (d 22 to 35 and 1 to 35) and serum levels of glucose and acute phase proteins (APPs). Regardless of temperature, the Ross 308 birds had significantly (p<0.05) lower IgA and higher finisher and overall BWG compared to Cobb 500.

**Conclusion:**

The present study suggests that the detrimental effects of heat stress are consistent across commercial broiler strains because there were no significant strain×temperature interactions for growth performance, serum APPs and immunoglobulin responses, meat quality, and ceacal microflora population.

## INTRODUCTION

Over the last few decades, considerable improvements in the growth rate of broiler chickens have been achieved. These improvements can be attributed to rapid advancements in genetic selection programs. However, heat stress remains a significant environmental challenge that can be financially costly for the poultry industry. The harmful consequences of heat stress stretch from a decreased body weight gain (BWG), feed intake (FI), and increased feed conversion ratio (FCR) of live birds [1], to substandard meat quality [2]. Furthermore, adverse effects on caecal microflora have been detected in heat-stressed broilers [3].

A variety of approaches for sustaining thermal tolerance of broiler chickens and reducing the harmful effects of heat stress are elicited [4]. An elevated level of glucose in chickens subjected to thermal challenges may enhance their survivability of chicken and promote maximal brain function. Creatine kinase (CK) is an enzyme that plays a role in the reversible transphosphorylation of adenosine diphosphate and creatine. In an earlier study [5], it was revealed that thermal stress increased the activity of blood CK. In related studies [6–8], it was found that heat stress can raise the level of serum in a group of proteins termed acute phase proteins (APPs). As such, these APPs, which include α1-acid glycoprotein (AGP), ovotransferrin (OVT), and ceruloplasmin (CPN), can be employed as indicators for assessing the physiological condition of chickens [9].

Immunoglobulin, which is generated by lymphocytes, rep resents a defence mechanism against the intrusion of foreign substances into the living body. Immunoglobulin adheres to the antigen (pathogen), which is subsequently swallowed up and digested by macrophages to shield the host from harm [10]. The three major immunoglobulin classes in chicken are immunoglobulin Y (IgY), IgM, and IgA. According to the results derived from previous studies, heat stress may decrease the relative weights of lymphoid organs in both laying hens [11], and broilers [12]. In an earlier investigation [13], it was revealed that broilers exposed to heat stress were negatively affected by a reduced level of total circulating antibodies.

The commensal intestinal microbiota significantly influ ences the digestion and absorption of nutrients, as well as the development of immunity [14]. The activities of commensal intestinal bacterial populations, also has a notable impact on the physiological and pathological conditions of the host. The adverse effects of heat stress on the intestinal mucosa and microbiota of broiler chickens and pigs have been verified through several investigations [1,15,16].

The influence of pre-slaughter stress reactions on ante and post-mortem muscle metabolism can lead to i) an increase in the speed and degree of glycogen breakdown, ii) a drop in pH, and iii) drip loss [17]. These effects of pre-slaughter stress are mainly attributed to alterations in the activities of adenosine triphosphate (ATP) and muscle glycogen reserves. Exposure to heat can have direct effects on organ and muscle metabolism [18]. For instance, the threats of pale-soft-exudative meat in turkeys, and heat shortening in broilers, can be promoted by heat stress. Also, operational alterations executed to counter the threats arising from heat exposure, can indirectly affect meat quality negatively [18]. Broilers that experienced an acute or short-term heat stress just before slaughter, displayed pale, soft, and exudative meat changes in the quality of their meat [19].

Cobb 500 and Ross 308 are the two most popular commer cial broiler strains. The former is an early developing strain while the latter is considered a late developing strain [20]. The resistance to heat stress in relation to different breeds has been thoroughly investigated [21]. A study on heat stress resistance involving jungle fowl, village fowl and commercial broiler chickens, revealed that domestication and selective breeding were resulting in birds that are more susceptible, rather than resistant, to heat stress [22]. However, information on the response of commercial broiler chicken strains towards a hot environment is still limited. As such, our investigation aims to determine the impact of normal and high temperatures on the growth performance, caecal microflora and immune response in two broiler strains.

## MATERIALS AND METHODS

### Ethical note

The protocol used in this experiment was carried out in full compliance with the Research Policy and Code of Practice for the Care and Use of Animal for Scientific Purposes of Universiti Putra Malaysia.

### Birds, housing and husbandry

Altogether, 120 Cobb 500 and 120 Ross 308 one-day-old male broiler chicks were obtained from a commercial hatchery. Subsequent to their weighing and wing banding, the chicks were randomly assigned by strain in groups of 10, in 24 battery cages. These cages, which come with wire floors, were arranged in 6 rooms. Each of these rooms, in which the environment is controllable, contained two cages of Cobb 500 and two cages of Ross 308 birds. The cages measured 122 cm in length, 91 cm in width and 61 cm in height. The initial room temperature was fixed at 32°C on d 1, and gradually lowered until it reached 23°C on d 21. Relative humidity extended from 70% to 80%. On d 7 and d 21, live Newcastle disease vaccine was administered to the birds.

### Diets

Both strains received the same commercial broiler starter (d 1 to 21) and finisher (d 22 to 35) diets. The feed was supplied from a local feed mill. The nutrient specifications of the diets are presented in Table 1. The starter (crumble form) and finisher (pellet form) diets were provided *ad libitum*. Metabolisable energies were 12.56 and 12.98 MJ/kg in the starter and finisher diets, respectively. Crude protein (CP) was 21.0% CP in the starter diet and 19.0% CP in the finisher diet.

### Heat challenge

In order to create a heat stress environment, 6 cages of birds from each strain (three rooms) were subjected to a temperature of 34°C from d 22 to d 35. This heat stress environment was prolonged for 6 hours each day. It is well documented that the heat challenge regimen [7,23,24], as measured by plasma corticosterone concentration, heterophil:lymphocyte ratio and heat shock protein 70 density, was stressful to broiler chickens. All throughout the heat challenge period, the regular temperature of 23°C was retained for the other rooms. This resulted in 4 subgroups from d 22 to d 35 (2 strains×2 temperatures).

### Growth rate parameters and sampling

On d 7, 14, 21, 28, and 35, the weights of the birds were recorded individually. The FIs were recorded on a weekly basis for a period of 5 weeks. The FI data was adjusted for mortality. FCR was calculated as feed/body weight; and the mortality rate was registered upon its occurrence. On d 35, two birds from every cage (12 birds for each strain-temperature subgroup) were picked at random for slaughter, which was conducted in compliance with halal procedures [25], which was performed humanely by severing the jugular veins, carotid arteries, trachea and oesophagus with a sharp knife by a single swipe in order to incur less pain. The exsanguination blood samples of these randomly selected birds were gathered and stored in ice. Soon after, these blood samples were centrifuged at 4,000×g for 20 minutes at 4°C. Serum samples were garnered, kept in storage at −80°C, and later tested for their concentrations of glucose, CK, APPs, IgY, IgA, and IgM. Subsequent to exsanguination, the caecal contents were immediately frozen in liquid nitrogen, and then stored at −80°C. Bacterial quantification analysis was later conducted on these caecal contents. The slaughtered birds’ entire *pectoralis major* breast meat was used for tests to determine the levels of pH, drip loss, and shear force.

### Glucose and creatine kinase

An automated chemistry analyser (Hitachi 902 Automatic Analyser; Hitachi, Tokyo, Japan) was harnessed to determine the concentration levels of serum CK and glucose. Cat. No.: 11447513 216 (glucose) and Cat. No.: 12132524 216 (CK) commercial kits (Roche Diagnostics, Basel, Switzerland) were utilized for this purpose.

### Acute phase proteins

The CPN concentration was determined by establishing the formation rate of a coloured product from CPN and the substrate, 1,4-phenylenediamine dihydrochloride, while OVT concentration was determined using a radial immune diffusion procedure, as described previously in detail [26]. A commercial enzyme-linked immunosorbent assay (ELISA) kit exclusively designed for chicken-related research (Cat. No. NB-E60049, Life Diagnostic Inc., West Chester, PA, USA) was used for measuring the serum AGP concentration.

### Immune response

The serum concentrations of IgY, IgM, and IgA were determined using commercial ELISA kits specific for chicken (Immunology Consultants Laboratory, Inc., Portland, OR, USA): chicken IgY (Cat. No. E-30Y), chicken IgA (Cat. No.E-30A), and chicken IgM (Cat. No. E-30M).

### Bacterial quantification

A quantitative real-time polymerase chain reaction (qPCR) [27] was used to determine the Lactobacilli, *Escherichia coli* (*E. coli*) and Clostridia populations. The extraction of deoxyribonucleic acid (DNA) from the caecal content samples was done by a QIAamp Fast DNA Stool Mini Kit (Qiagen Inc., Valencia, CA, USA). While the purity and concentration of these DNA samples were measured with the use of a Nanodrop ND-1000 spectrophotometer, their purification was done by MEGA quick-spinTM (Intron Biotechnology, Inc., Seongnam, Korea). The total count of DNA replications for each mL of the elution buffer was appropriately computed. The structuring of standard curves was achieved through serial dilution of the PCR products from pure cultures of Lactobacilli, *E. coli*, and Clostridia. The primers utilized for bacterial quantification were as follows: Lactobacilli (F-5 CATCCAG TGCAAACCTAAGAG-3 and R-5 GATCCGCTTGCCTT CGCA-3), *E. coli* (F-50-GTGTGATATCTACCCGCTTCGC-3 and R-50-AGAACGCTTTGTGGTTAATCAGGA-3), and Clostridia (F-5 GAGTTTGATCMTGGCTCAG-3 and R-5 CCCTTTACACCCAGTAA-3). Optical grade plates were used to execute the q-PCR with BioRad CFX96 Touch (BioRad, Hercules, CA, USA). The PCR was performed in 25 μL of total volume. Each reaction involved 12.5 μL Maxima SYBER Green qPCR Master Mix (Thermo Fisher Scientific, Waltham, MA, USA), 1 μL of each primer, 1 μL of the DNA samples, and 9.5 μL of nuclease-free H_2_O. The amplification reaction stipulations of the DNA were 94°C for a period of 5 min, then 40 cycles of 94°C for a period of 20 s, 58°C (Lactobacilli), or 60°C (Clostridia and *E. coli*) for a period of 30 s, and 72°C for a period of 20 s. Subsequent to every final amplification cycle, a melting curve analysis was conducted to verify the particularity of the amplification.

### Meat quality

#### Muscle pH

The samples to determine muscle pH were taken from the right *pectoralis major*. The initial pH (pH_i_) was recorded 45 minutes after slaughter, while the ultimate pH (pH_u_) was recorded after 24 hours of chilling. This procedure is in accordance with that modified by Abdulla et al [28]. Roughly 0.5 g of meat sample was first homogenized in 10 mL of 4°C distilled water. Subsequently, a pH meter furnished with an electrode was used for the measurement of pH (Mettler Toledo, Columbus, OH, USA).

#### Drip loss

Samples for the determination of drip loss were obtained from the left *pectoralis major* following the procedure recommended by Honikel [29]. Directly after slaughter, the weights of the separately weighed meat samples were registered as the initial weight (W1). The samples were then consigned to a sealed, vacuumed polyethylene bag and stored at 4°C. After 24 hours, the samples were removed from the bags, blot dried with the use of soft tissue paper, and re-weighed (W2). The equation below was used to calculate the drip loss percentage of muscle chilled over 24 hours:

Drip loss (%)=[(W1-W2)/W1]×100

#### Shear force

Samples from the drip loss determination were used to measure the texture of the sample via shear force analysis. Following the determination of the drip loss percentage, these samples were shifted into polyethylene bags, and immersed in a water bath set at a temperature of 80°C. After a period of 30 minutes, the samples were taken out of the water bath and left to cool down to room temperature. Upon arrival at room temperature, the samples were sliced into three sub-samples measuring 10 cm in width, 1 cm in height and 2 cm in length. In order to ascertain the shear force value of each sub-sample, a Volodkevitch bite jaw, fastened to a TA.HD plus texture analyser (Stable Micro System, Surrey, UK) equipped with a 5 kg load cell, was brought into play. The average of the three sub-sample values was calculated, and registered as the shear force values.

### Statistical analysis

The data were subjected to analysis of variance by way of the general linear model procedure of SAS software (SAS Institute Inc. Cary, NC, USA). The FI, BWG, and FCR data from d 1 to 21 were analysed with the strain as the only main effect. The FI, BWG, and FCR data from d 22 to 35 and d 1 to 35, glucose, CK, APPs, immune response, caecal microflora, and meat quality data were analysed using strain, temperature, and their interactions as main effects. Comparisons were made within each experimental variable, whenever the interactions between the effects were observed to be significant. Duncan’s multiple range test was employed to determine the differences between means. Chi-square analysis was applied for the mortality data, and the statistical significance was considered at p<0.05.

## RESULTS

### Growth rate and mortality

Throughout the starter phase, strain did not have any impact on FI, BWG, or FCR (Table 1). Throughout the heat exposure period, no notable interactions were perceived between strain and temperature for FI, BWG, or FCR during the finisher and overall (d 1 to 35) phases (Table 2). During the finisher and overall phases, the heat exposure significantly (p<0.01) decreased FI, BWG, and FCR. Irrespective of temperature, the Ross 308 birds displayed a significantly (p<0.05) higher BWG than Cobb 500 during the finisher and overall phases. Throughout the finisher and overall phases, FI and FCR were not influenced by strain. All through the heat exposure phase, the mortality rate was not affected by strain, but it was considerably (p<0.05) higher in heat challenged birds than in their non-heat challenged counterparts.

### Physiological stress indicators

Significant interactions were noted between strain and temperature for serum concentration of CK, but not for glucose, AGP, CPN, or OVT (Table 3). At 35 d of age, heat stress resulted in significantly (p<0.05) higher CK within the Cobb 500 broiler chickens, but not within Ross 308 (Table 4). Regardless of temperature, strain had no significant effect on glucose, AGP, CPN, and OVT concentrations (Table 3). Serum concentrations of glucose, AGP, CPN, and OVT were greater (p<0.01) in heat stressed birds than in their non-heat stressed counterparts (Table 3).

### Immune response

No interactions were noticeable between strain and temperature for IgY, IgM, or IgA (Table 5). At the age of 35 days, the Cobb 500 chickens exhibited a considerably higher (p<0.01) serum concentration of IgA than Ross 308. No strain effect was detected for concentrations of IgM and IgY. At 35 days of age, heat stress substantially (p<0.01) reduced the concentrations of IgY and IgM, but not that of IgA.

### Caecal microbial population

No interactions were detected between strain and temperature for caecal populations of Lactobacilli, *E. coli* or Clostridia (Table 6). Both strain and temperature did not have any impact (p>0.05) on the quantified caecal microbial populations.

### Meat quality measurements

No significant interactions were observed between strain and temperature for breast muscle pH_i_, pH_u_, drip loss or shear force in broiler chickens at 35 d of age (Table 7). Strain had no effect on breast muscle pH_i_, pH_u_ or drip loss. However, strain significantly (p<0.05) affected the shear force value as Ross 308 broilers showed higher meat shear force than Cobb 500. Regardless of strain, heat stress greatly decreased (p<0.01) pH_i_ and pH_u_, and increased (p<0.05) shear force value compared to normal temperature. Temperature did not have any effect on drip loss.

## DISCUSSION

In the present study we evaluated the growth performance, gut microbiota and immune response of two common commercial broiler strains, Cobb 500 and Ross 308 under heat stress conditions. The current results indicated that there were no differences in FI, BWG, or FCR between the two strains before the heat stress exposure (1 to 21 d of age). However, during the heat stress period (22 to 35 d of age), the Ross 308 birds showed significantly higher BWG compared to Cobb 500 during both the finisher and overall phases without affecting the FCR or FI. The noted improved BWG in the former during the finisher and overall phases could also be attributed to nutritional factor. Ross 308 and Cobb 500 broilers have different metabolizable energy and CP requirements [30,31]. However, in this study, to mimic the commercial production setting, both Ross 308 and Cobb 500 broilers were fed similar commercial starter and finisher diets. Tona et al [32] who found that at 6 and 60 h post hatch, heat production was higher in Cobb than in Ross. The authors concluded that Cobb and Ross embryos-chicks had different growth trajectories leading to different patterns of growth resulting from physiological variations. However, our current results suggest that there were no genetic differences between Ross 308 and Cobb 500 in response to heat stress conditions. Working with various levels of dietary lysine, Sterling et al [33] reported that Cobb broiler chicks gained more weight, consumed higher feed, and had a better FCR when compared with Ross 308 broiler chicks. In the present study, in terms of BWG, there were no interactions between strain and temperature during both the finisher and overall phases. This suggests that irrespective of temperature, the growth rate of Ross 308 broilers is superior to that of Cobb 500 broilers. The discrepancies between the present findings and those of Sterling et al [33] could be attributed to the genetic disparities between the present day Ross 308 strain, and those of 2006.

Unsurprisingly, heat stress adversely affects the FI, body weight, and FCR of broiler chickens. The issues related to reduced feed consumption and weight gain, in terms of broilers reared in a stifling environment, are well documented in relevant literature [34]. The inferior growth performance of broilers subjected to heat stress conditions can be due to the behavioural, metabolic, and physiological alterations in reaction to a hot environment. Other than the decline in FI, respiratory alkalosis can also be held accountable for the drop in growth rate under heat stress [35]. Moreover, the greater dispersal of energy by birds subjected to heat stress can also lead to their unsatisfactory growth rate [36]. Mortality rate during the heat treatment was not affected by strain, but the heat challenge was detrimental to the survivability rate. The higher mortality rate during heat stress is expected, and confirmed the association between the high environmental temperatures and the increase in mortality rate in broilers [34].

Stress triggers the liberation of catecholamines and gluco corticoids in birds. This brings about alterations in biochemical variables (such as glucose) aimed at maintaining homeostasis [37]. During our investigation, it was observed that heat stress caused an increase in the levels of blood glucose. This observation is in agreement with that of Akşit et al [38]. A rise in the level of glucocorticoids contributes directly towards an elevation in the concentration of blood glucose [39]. Glucocorticoids significantly influence metabolism by inciting gluconeogenesis from muscle tissue proteins, lymphoid and connective tissues. They also dictate many aspects of glucose homeostasis. The main role of glucocorticoids, in the context of glucose homeostasis, is the conservation of plasma glucose for the brain during a stressful situation. This is important as transitorily higher blood pressure is essential for the achievement of optimal brain function [40]. The CK is a fundamental enzyme for the continued supply of intracellular energy through the spatio-temporal buffering of ATP concentrations [41]. An increase in plasma CK is indicative of skeletal muscle damage or myopathy, the consequence of disruptions in muscle cell membrane (sarcolemma) function and permeability [42]. In our study, we detected noteworthy interactions between strain and temperature for CK. These interactions became evident when the heat stressed Cobb 500 birds exhibited substantially greater CK than their non-heat stressed counterparts. On the contrary, temperature did not significantly influence CK in Ross 308 broilers. These results suggest that, Cobb 500 birds were more susceptible to heat stress than Ross 308. According to the present results, there was a significant elevation in serum concentrations of AGP, CPN, and OVT following heat exposure. These findings are in accord with recent studies indicating that heat stress elevates serum levels of APPs in broilers [6–8], and thus APPs are reliable stress biomarkers in broilers. The goal of the APPs is to re-establish homeostasis. While AGP is an established immunoregulator that influences T-cell performance and conveys negative feedback on the acute phase response, CPN portrays a more shielding role by its removal of oxygen radicals, its antihistamine activity, and its reversal of the hypoferraemic state of the APR [43]. The OVT, an iron binding protein, can impart antimicrobial properties by requisitioning iron. This protein also comes with the capacity to adjust the heterophil and macrophage roles in chickens. Thus, the reaction of APPs can be deemed part of the overall physiological stress response, which involves the hypothalamic-pituitary-adrenal axis and the sympathetic system. Leshchinsky and Klasing [44] compared APPs response to disease challenge in broiler and layer chickens and concluded that the latter showed a better reaction. During our investigation, it was established that the serum levels of AGP, CPN, and OVT are identical for the Cobb 500 and Ross 308 strains. The prominent disparities between broilers and layers, as identified by Leshchinsky and Klasing [44], imply that discrepancy in the rate of growth may influence the response of APPs in poultry.

Under commercial broiler management conditions, fast-growing broilers exhibited high mortality from commonly encountered infectious or metabolic diseases when compared with slower growing groups of birds [45]. The present study indicated that Cobb 500 chickens had a higher serum level of IgA than Ross 308 while strain had a negligible effect on concentrations of IgM and IgY on d 35. This suggests that IgA appears to be less sensitive to the heat challenge than IgM and IgY. According to Cheema et al [46], genetic selection for a better broiler performance, resulted in an unfavourable effect on the adaptive element of the immune response (antibody generation). Our current results further support the study by Cheema et al [46] who reported higher weight gains in Ross 308 was associated with lower serum IgA levels than those of Cobb 500. According to our results, the heat challenge significantly reduced the levels of IgY and IgM, but not IgA on d 35. These results confirm the claim from previous studies [47–49] that heat stress damages the immune response in poultry.

The commensal intestinal bacterial populations also play an important pathological effect on the host [14]. This intricate ecosystem achieves this by i) contending for epithelial binding locations and nutrients, ii) reinforcing the intestinal immune response, and iii) generating antimicrobial bacteriocins [15]. Song et al [3] reported lower counts of *Lactobacillus* spp. and higher counts of *Clostridium* spp. in heat-stressed broilers. Contrastingly, our results indicated that the effect of temperature on caecal populations of Lactobacilli, *E. coli* or Clostridia, is minimal. This contradiction may be due to differences in the harshness and time span of these heat endurance exercises. While Song et al [3] exposed broilers to a temperature of 33°C for 10 h each day from d 22 to d 42, our study involved a temperature of 34°C for 6 h each day from d 22 to d 35. The genetic alterations related to enhanced weight gain and FI, also led to transformations in the birds’ gut physiology and microbial population make-up [50]. Gut microflora profoundly improves the nutrition, health and growth performance of its host [51]. Our investigations revealed that the caecal populations of Lactobacilli, *E. coli*, and Clostridia, were the same for Ross 308 and Cobb 500 birds. Thus, it appears that the greater weight gain in Ross 308 birds when compared to their Cobb 500 counterparts was not associated with intestinal microflora.

Heat stress, feed retraction, conveyance and liquid depri vation, are among pre-slaughter stressors that can affect meat quality [52]. Pre-slaughter heat stress can quicken the development of rigor mortis, which encourages a rapid pH decline, a lower pH_u_, and an increased lightness value in bird meat. The ultimate result is chicken/turkey meat with pale, exudative features [19,53]. Generally, muscle pH is roughly 7, and descends quickly subsequent to slaughter. In our study, the meat pH 24 hours post-slaughter arrived at an average of 5.90 to 6.09. According to our results, while strain had no distinct effect on meat pH values, heat exposure drastically decreased pH_i_ and pH_u_ values. This could be linked to disparities in the glycogen stores of the muscle, or disparities in the glycolytic possibilities between heat enduring and non-heat enduring situations. It appears that the greater pH fall was caused by the higher rate of post mortem glycolysis which led to the higher production of lactate, and the lowered ultimate pH in the meat of broilers. Breast meat pH_u_ measurements 24 hours after chilling at 4°C revealed an overall decline in pH. The more pronounced reduction in pH_u_ in comparison to pH_i_, could be due to the further extent of post mortem glycolytic metabolism in more aged muscles [54]. The retention and gains or losses of water, need to be seriously considered as they affect the weight of the chicken, and consequently, its economic value. Also, the amount and manner of water dissemination in muscles play a prominent role in determining the meat’s appearance, tenderness and succulence. Previous studies have indicated that severe and persistent heat stresses can lead to mediocre water-holding properties [53,55]. The elevated metabolic rate of rigor mortis associated with heat stress, causes marked protein denaturation in muscles [56,57]. This condition reduces the protein’s capacity for water-binding, which consequently culminates in unsatisfactory water-holding properties [58]. Lu et al [59] stated that drip loss is obvious in the muscles of heat-stressed Arbor Acres broilers. In our investigation, however, both Cobb 500 and Ross 308 strains showed a negligible effect on drip loss during heat endurance. These conflicting outcomes could be due to disparities in the intensity of the heat challenge. While the birds in our study were subjected to a temperature of 34°C for 6 hours daily from d 21 to d 35, the birds in the experiment by Lu et al [59] were exposed to constant heat stress conditions. In our present results, it was observed that shear force (a measure of meat tenderness) is substantially greater in the meat of heat stressed birds, than in their non-heat stressed counterparts. This outcome is in harmony with that of Dai et al [60] who found that shear force is greater in the breast meat of heat stressed birds, than in the breast meat of control broilers raised in a normal temperature. Heat stress can induce liquid outflow from muscle, loss of soluble nutrients and flavour. This is likely to render the muscle dry, hard and tasteless [60].

## CONCLUSION

In conclusion, considering the lack of significant strain× temperature interactions for all the parameters measured, except for CK, the Cobb 500 and Ross 308 strains did not differ in any major way in response to heat stress. However, irrespective of temperature, the Ross 308 showed higher BWG during the finisher phase and higher serum IgA concentration on day 35 than their Cobb 500 counterparts.

## Figures and Tables

**Table 1:** Nutrient specifications of experimental diets1)

Nutrient (% unless stated otherwise)	Starter (d 1–21)2)	Finisher (d 22–35)3)
Metabolisable energy (MJ/kg)	12.56	12.98
Crude protein	21.0	19.0
Crude fibre	5.00	5.00
Crude fat	4.50	5.00
Moisture	13.0	13.0
Ash	8.00	8.00
Calcium	0.80	0.80
Available phosphorus	0.40	0.40
Digestible lysine	1.20	1.08
Digestible methionine+cysteine	0.85	0.78

1) Diets were formulated using the following ingredients: corn, soybean meal, other grains and grain-by-product, animal protein, vegetable oil, salt, calcium carbonate, coccidiostats and approved antimicrobials.

2) Crumble form.

3) Pellet form.

**Table 2:** Effects of strain and temperature on feed intakes, body weights, feed conversion ratios, and mortality rate in broiler chickens from 1 to 35 days of age

Variable	Strain		SEM	Temp1)		SEM	p-value		
		
	Cobb 500	Ross 308		Normal	Heat stress		Strain	Temp	Strain×Temp
Feed intake (g/bird)									
Day 1 to 21	1,287	1,285	48.0	NA	NA	-	0.904	NA	NA
Day 22 to 35	2,281	2,339	167	2,418^a^	2,202^b^	127	0.269	<0.001	0.319
Day 1 to 35	3,568	3,624	151	3,697^a^	3,494^b^	112	0.236	<0.001	0.524
Body weight gain (g)									
Day 1 to 21	1,044	1,042	36.0	NA	NA	-	0.932	NA	NA
Day 22 to 35	1,301^b^	1,372^a^	147	1,461^a^	1,210^b^	76.7	0.018	<0.001	0.205
Day 1 to 35	2,345^b^	2,414^a^	137	2,495^a^	2,263^b^	72.7	0.018	<0.001	0.512
FCR (feed/gain)									
Day 1 to 21	1.23	1.23	0.03	NA	NA	-	0.988	NA	NA
Day 22 to 35	1.75	1.70	0.11	1.66^b^	1.82^a^	0.08	0.145	<0.001	0.993
Day 1 to 35	1.52	1.50	0.05	1.48^b^	1.54^a^	0.04	0.211	<0.001	0.872
Mortality (Day 22 to 35)	21.6	19.2	12.4	10.0^b^	31.4^a^	8.60	-	-	-

SEM, standard error of the mean for main effects (n = 12); Temp, temperature; NA, not applicable; FCR, feed conversion ratios.

1) The heat stressed birds were exposed to 34°C for 6 h daily.

a,b Means within a row-subgroup with no common superscripts are significantly different at p<0.05.

**Table 3:** Effects of strain and temperature on serum concentrations of glucose, creatine kinase, α1-acid glycoprotein, ceruloplasmin, and ovotransferrin in broiler chickens at 35 days of age

Variable	Strain		SEM	Temp1)		SEM	p-value		
		
	Cobb 500	Ross 308		Normal	Heat stress		Strain	Temp	Strain×Temp
Glucose (mmol/L)	21.96	21.18	3.18	20.13^b^	23.00^a^	2.85	0.348	0.001	0.686
Creatine kinase (mmol/L)	30.17	29.58	4.92	28.75	31.00	4.79	0.666	0.101	0.035
AGP (mg/mL)	0.571	0.546	0.19	0.460^b^	0.656^a^	0.17	0.614	<0.001	0.852
CPN (mg/mL)	4.493	4.413	0.89	4.001^b^	4.906^a^	0.76	0.719	<0.001	0.353
OVT (mg/mL)	1.456	1.508	0.18	1.374^b^	1.589^a^	0.17	0.309	<0.001	0.861

SEM, standard error of the mean for main effects (n = 24); Temp, temperature; AGP, α1-acid glycoprotein; CPN, ceruloplasmin; OVT, ovotransferrin.

1) The heat stressed birds were exposed to 34°C for 6 h daily.

a,b Means within a row-subgroup with no common superscripts are significantly different at p<0.05.

**Table 4:** Creatine kinase (mmol/L) when the interactions between strain and temperature were significant

Strain	Temperature		SEM	p-value

	Normal	Heat stress		
Cobb 500	27.58^b^	32.75^a^	5.16	0.023
Ross 308	29.92	29.25	4.09	0.693
SEM	4.68	4.62	-	-
p-value	0.235	0.077	-	-

SEM, standard error of the mean for strain effect (n = 12) and temperature effect (n = 12).

a,b Means within a row with no common superscripts are significantly different at p<0.05.

**Table 5:** Effects of strain and temperature on serum concentrations of IgY, IgM, and IgA in broiler chickens at 35 days of age

Variable	Strain		SEM	Temp1)		SEM	p-value		
		
	Cobb 500	Ross 308		Normal	Heat stress		Strain	Temp	Strain×Temp
IgY (mg/mL)	2.69	2.60	0.82	2.07^b^	3.22^a^	0.57	0.588	<0.001	0.413
IgM (mg/mL)	0.29	0.27	0.09	0.22^b^	0.35^a^	0.07	0.366	<0.001	0.980
IgA (mg/mL)	0.25^a^	0.19^b^	0.05	0.23	0.22	0.06	0.003	0.767	0.866

Ig, immunoglobulin; SEM, standard error of the mean for main effects (n = 24); Temp, temperature.

1) The heat stressed birds were exposed to 34°C for 6 h daily.

a,b Means within a row-subgroup with no common superscripts are significantly different at p<0.05.

**Table 6:** Effects of strain and temperature on caecal Lactobacilli, *Escherichia coli*, and Clostridia populations in broiler chickens at 35 days of age

Variable	Strain		SEM	Temp1)		SEM	p-value		
		
	Cobb 500	Ross 308		Normal	Heat stress		Strain	Temp	Strain×Temp
Lactobacilli (log_10_ cfu/g)	6.69	6.73	0.54	6.63	6.79	0.53	0.694	0.147	0.882
*Escherichia coli* (log_10_ cfu/g)	5.80	5.79	0.71	5.83	5.76	0.71	0.922	0.643	0.593
Clostridia (log_10_ cfu/g)	7.42	7.49	0.60	7.54	7.38	0.59	0.503	0.194	0.759

SEM, standard error of the mean for main effects (n = 24); Temp, temperature.

1) The heat stressed birds were exposed to 34°C for 6 h daily.

**Table 7:** Effects of strain and temperature on breast muscle initial pH (pH_i_), ultimate pH (pH_u_), drip loss, and shear force in broiler chickens at 35 days of age

Variable	Strain		SEM	Temp1)		SEM	p-value		
		
	Cobb 500	Ross 308		Normal	Heat stress		Strain	Temp	Strain×Temp
pH_i_	6.44	6.41	0.15	6.66^a^	6.19^b^	0.12	0.384	<0.001	0.303
pH_u_	5.99	6.03	0.14	6.09^a^	5.93^b^	0.13	0.459	<0.001	0.608
Drip loss (%)	1.03	1.18	0.64	1.12	1.08	0.60	0.398	0.832	0.224
Shear force (N)	1,094^b^	1,270^a^	163	1,093^b^	1,276^a^	151	0.015	0.013	0.731

SEM, standard error of the mean for main effects (n = 24); Temp, temperature.

1) The heat stressed birds were exposed to 34oC for 6 h daily.

a,b Means within a row-subgroup with no common superscripts are significantly different at p<0.05.

## References

[1] Awad EA, Idrus Z, Soleimani Farjam A, Bello AU, Jahromi MF (2018). Growth performance, duodenal morphology and the caecal microbial population in female broiler chickens fed glycine-fortified low protein diets under heat stress conditions. Br Poult Sci.

[2] Sifa D, Bai X, Zhang D (2018). Dietary glutamine improves meat quality, skeletal muscle antioxidant capacity and glutamine metabolism in broilers under acute heat stress. J Appl Anim Res.

[3] Song J, Xiao K, Ke YL (2014). Effect of a probiotic mixture on intestinal microflora, morphology, and barrier integrity of broilers subjected to heat stress. Poult Sci.

[4] Gonzalez-Esquerra R, Leeson S (2006). Physiological and metabolic responses of broilers to heat stress-implications for protein and amino acid nutrition. Worlds Poult Sci J.

[5] Hocking PM, Maxwell MH, Mitchell MA (1994). Haematology and blood composition at two ambient temperatures in genetically fat and lean adult broiler breeder females fed *ad libitum* or restricted throughout life. Br Poult Sci.

[6] Najafi P, Zulkifli I, Jajuli NA (2015). Environmental temperature and stocking density effects on acute phase proteins, heat shock protein 70, circulating corticosterone and performance in broiler chickens. Int J Biometeorol.

[7] Zulkifli I, Akmal AF, Soleimani AF, Hossain MA, Awad EA (2018). Effects of low-protein diets on acute phase proteins and heat shock protein 70 responses, and growth performance in broiler chickens under heat stress condition. Poult Sci.

[8] Law FL, Zulkifli I, Soleimani AF, Liang JB, Awad EA (2019). Effects of protease supplementation of low protein and/or energy diets on growth performance and blood parameters in broiler chickens under heat stress condition. Ital J Anim Sci.

[9] O’reilly EL, Eckersall PD (2014). Acute phase proteins: a review of their function, behaviour and measurement in chickens. Worlds Poult Sci J.

[10] Ohashi Y, Hiraguchi M, Ushida K (2006). The composition of intestinal bacteria affects the level of luminal IgA. Biosci Biotechnol Biochem.

[11] Ghazi SH, Habibian M, Moeini MM, Abdolmohammadi AR (2012). Effects of different levels of organic and inorganic chromium on growth performance and immunocompetence of broilers under heat stress. Biol Trace Elem Res.

[12] Niu ZY, Liu FZ, Yan QL, Li WC (2009). Effects of different levels of vitamin E on growth performance and immune responses of broilers under heat stress. Poult Sci.

[13] Bartlett JR, Smith MO (2003). Effects of different levels of zinc on the performance and immunocompetence of broilers under heat stress. Poult Sci.

[14] Leser TD, Mølbak L (2009). Better living through microbial action: the benefits of the mammalian gastrointestinal microbiota on the host. Environ Microbiol.

[15] Burkholder KM, Thompson KL, Einstein ME, Applegate TJ, Patterson JA (2008). Influence of stressors on normal intestinal microbiota, intestinal morphology, and susceptibility to *Salmonella* enteritidis colonization in broilers. Poult Sci.

[16] Pearce SC, Mani V, Boddicker RL (2013). Heat stress reduces intestinal barrier integrity and favors intestinal glucose transport in growing pigs. PloS One.

[17] Terlouw C (2005). Stress reactions at slaughter and meat quality in pigs: genetic background and prior experience: a brief review of recent findings. Livest Prod Sci.

[18] Gregory NG (2010). How climatic changes could affect meat quality. Food Res Int.

[19] Northcutt JK, Foegeding EA, Edens FW (1994). Water-holding properties of thermally preconditioned chicken breast and leg meat. Poult Sci.

[20] Berhe ET, Gous RM (2005). Effect of dietary protein content on allometric relationships between carcass portions and body protein in Cobb and Ross broilers.

[21] Daghir NJ (2008). Poultry production in hot climates.

[22] Soleimani AF, Zulkifli I, Omar AR, Raha AR (2011). Physiological responses of 3 chicken breeds to acute heat stress. Poult Sci.

[23] Ramiah SK, Awad EA, Mookiah S, Idrus Z (2019). Effects of zinc oxide nanoparticles on growth performance and concentrations of malondialdehyde, zinc in tissues, and corticosterone in broiler chickens under heat stress conditions. Poult Sci.

[24] Olubodun J, Zulkifli I, Hair-Bejo M, Kasim A, Soleimani AF (2015). Physiological response of glutamine and glutamic acid supplemented broiler chickens to heat stress. Eur Poult Sci.

[25] Farouk MM, Al-Mazeedi HM, Sabow AB (2014). Halal and kosher slaughter methods and meat quality: a review. Meat Sci.

[26] Zulkifli I, Najafi P, Nurfarahin AJ (2014). Acute phase proteins, interleukin 6, and heat shock protein 70 in broiler chickens administered with corticosterone. Poult Sci.

[27] Bello AU, Idrus Z, Yong Meng G, Awad EA, Soleimani AF (2018). Gut microbiota and transportation stress response affected by tryptophan supplementation in broiler chickens. Ital J Anim Sci.

[28] Abdulla NR, Mohd Zamri AN, Sabow AB (2017). Physico-chemical properties of breast muscle in broiler chickens fed probiotics, antibiotics or antibiotic–probiotic mix. J Appl Anim Res.

[29] Honikel KO (1998). Reference methods for the assessment of physical characteristics of meat. Meat Sci.

[30] Cobb (2015). Cobb 500 broiler performance and nutrition supplement.

[31] Ross (2014). Ross 308 broiler: Nutrition specifications.

[32] Tona K, Onagbesan OM, Kamers B, Everaert N, Bruggeman V, Decuypere E (2010). Comparison of Cobb and Ross strains in embryo physiology and chick juvenile growth. Poult Sci.

[33] Sterling KG, Pesti GM, Bakalli RI (2006). Performance of different broiler genotypes fed diets with varying levels of dietary crude protein and lysine. Poult Sci.

[34] Lara LJ, Rostagno MH (2013). Impact of heat stress on poultry production. Animals.

[35] Leeson S (1986). Nutritional considerations of poultry during heat stress. Worlds Poult Sci J.

[36] Sohail MU, Hume ME, Byrd JA (2012). Effect of supplementation of prebiotic mannan-oligosaccharides and probiotic mixture on growth performance of broilers subjected to chronic heat stress. Poult Sci.

[37] Remage-Healey L, Romero LM (2001). Corticosterone and insulin interact to regulate glucose and triglyceride levels during stress in a bird. Am J Physiol Regul Integr Comp Physiol.

[38] Akşit M, Yalcin S, Özkan S, Metin K, Özdemir D (2006). Effects of temperature during rearing and crating on stress parameters and meat quality of broilers. Poult Sci.

[39] Borges SA, Fischer da Silva AV, Majorka A, Hooge DM, Cummings KR (2004). Physiological responses of broiler chickens to heat stress and dietary electrolyte balance (sodium plus potassium minus chloride, milliequivalents per kilogram). Poult Sci.

[40] Kuo T, McQueen A, Chen T-C, Wang J-C, Wang JC, Harris C (2015). Regulation of glucose homeostasis by glucocorticoids. Glucocorticoid signaling from molecules to mice to man.

[41] Clark JF, Khuchua Z, Kuznetsov A, Saks VA, Ventura-Clapier R (1993). Compartmentation of creatine kinase isoenzymes in myometrium of gravid guinea-pig. J Physiol.

[42] Mitchell MA, Sandercock DA (1995). Creatine kinase isoenzyme profiles in the plasma of the domestic fowl (*Gallus domesticus*): effects of acute heat stress. Res Vet Sci.

[43] Murata H, Shimada N, Yoshioka M (2004). Current research on acute phase proteins in veterinary diagnosis: an overview. Vet J.

[44] Leshchinsky TV, Klasing KC (2001). Divergence of the inflammatory response in two types of chickens. Dev Comp Immunol.

[45] Yunis R, Ben-David A, Heller ED, Cahaner A (2000). Immunocompetence and viability under commercial conditions of broiler groups differing in growth rate and in antibody response to *Escherichia coil* vaccine. Poult Sci.

[46] Cheema MA, Qureshi MA, Havenstein GB (2003). A comparison of the immune response of a 2001 commercial broiler with a 1957 randombred broiler strain when fed representative 1957 and 2001 broiler diets. Poult Sci.

[47] Kidd MT (2004). Nutritional modulation of immune function in broilers. Poult Sci.

[48] Mashaly MM, Hendricks GL, Kalama MA, Gehad AE, Abbas AO, Patterson PH (2004). Effect of heat stress on production parameters and immune responses of commercial laying hens. Poult Sci.

[49] Zulkifli I, Norbaiyah B, Nor Azah AS (2004). Growth performance, mortality and immune response of two commercial broiler strains subjected to early age feed restriction and heat conditioning under the hot, humid tropical environment. Arch Geflugelk.

[50] Lumpkins BS, Batal AB, Lee MD (2010). Evaluation of the bacterial community and intestinal development of different genetic lines of chickens. Poult Sci.

[51] Yang Y, Iji PA, Choct M (2009). Dietary modulation of gut microflora in broiler chickens: a review of the role of six kinds of alternatives to in-feed antibiotics. Worlds Poult Sci J.

[52] González VA, Rojas GE, Aguilera AE (2007). Effect of heat stress during transport and rest before slaughter, on the metabolic profile, blood gases and meat quality of quail. Int J Poult Sci.

[53] McKee SR, Sams AR (1997). The effect of seasonal heat stress on rigor development and the incidence of pale, exudative turkey meat. Poult Sci.

[54] Ismail SN, Awad EA, Zulkifli I, Goh YM, Sazili AQ (2019). Effects of method and duration of restraint on stress hormones and meat quality in broiler chickens with different body weights. Asian-Australas J Anim Sci.

[55] Molette C, Rémignon H, Babilé R (2003). Maintaining muscles at a high post-mortem temperature induces PSE-like meat in turkey. Meat Sci.

[56] Van Laack RLJM, Liu C-H, Smith MO, Loveday HD (2000). Characteristics of pale, soft, exudative broiler breast meat. Poult Sci.

[57] Gotardo LRM, Vieira PB, Marchini CFP (2015). Cyclic heat stress in broilers and their effects on quality of chicken breast meat. Acta Sci Vet.

[58] Honikel KO, Everts ME, Pas MFWt, Haagsman HP (2004). Water-holding capacity of meat. Muscle development of livestock animals: physiology, genetics and meat quality.

[59] Lu Q, Wen J, Zhang H (2007). Effect of chronic heat exposure on fat deposition and meat quality in two genetic types of chicken. Poult Sci.

[60] Dai SF, Wang LK, Wen AY, Wang LX, Jin GM (2009). Dietary glutamine supplementation improves growth performance, meat quality and colour stability of broilers under heat stress. Br Poult Sci.

